# Tree peony transcription factor *PrWRI1* enhances seed oil accumulation

**DOI:** 10.1186/s12870-023-04127-9

**Published:** 2023-03-07

**Authors:** Lihang Xie, Jiayuan Hu, Zhenguo Yan, Xinyao Li, Sailong Wei, Ruilin Xu, Weizong Yang, Huihui Gu, Qingyu Zhang

**Affiliations:** 1 Academy of Medical Sciences, Zhengzhou University, Zhengzhou, 450000 Henan China; 2 Sichuan Academy of Giant Panda, Chengdu, 610000 Sichuan China; 3 Academy of Agricultural Planning and Engineering, MARA, Beijing, 100000 China; 4 School of Life Sciences, Zhengzhou University, Zhengzhou, 450000 Henan China; 5 College of Landscape Architecture and Art, Northwest A&F University, Yangling, 712100 Shannxi China

**Keywords:** *Paeonia rockii*, AP2 transcription factor, *PrWRI1*, Seeds oil, Fatty acid biosynthesis

## Abstract

**Background:**

WRINKLED1 (*WRI1*) encodes a transcription factor, belonging to the APETALA2 (AP2) family, and plays a key role in regulating plant oil biosynthesis. As a newly woody oil crop, tree peony (*Paeonia rockii*) was notable for the abundant unsaturated fatty acids in its seed oil. However, the role of *WRI1* during the accumulation of *P. rockii* seeds oil remains largely unknown.

**Results:**

In this study, a new member of the *WRI1* family was isolated from *P. rockii* and was named *PrWRI1*. The ORF of *PrWRI1* consisted of 1269 nucleotides, encoding a putative protein of 422 amino acids, and was highly expressed in immature seeds. Subcellular localization analysis in onion inner epidermal cells showed that *PrWRI1* was located at the nucleolus. Ectopic overexpression of *PrWRI1* could significantly increase the total fatty acid content in *Nicotiana benthamiana* leaf tissue and even PUFAs in transgenic *Arabidopsis thaliana* seeds. Furthermore, the transcript levels of most genes related to fatty acids (FA) synthesis and triacylglycerol (TAG) assembly were also up-regulated in transgenic *Arabidopsis* seeds.

**Conclusions:**

Together, *PrWRI1* could push carbon flow to FA biosynthesis and further enhance the TAG amount in seeds with a high proportion of PUFAs.

**Supplementary Information:**

The online version contains supplementary material available at 10.1186/s12870-023-04127-9.

## Background

Fatty acids are important structural components for cells and provide the necessary energy for human beings. According to the presence or absence of double bonds in its long aliphatic chain, fatty acids could be classified into two major groups, including saturated and unsaturated fatty acids (UFAs). There is growing scientific evidence showing that the supply of UFAs, especially *n*-3 polyunsaturated fatty acids (PUFAs) could reduce the risk of coronary heart disease [1, 2]. The most common *n*-3 PUFAs in plant oil are *α*-linolenic acid (ALA). ALA is mainly found in canola (5%), soybean (2%), and walnut oils (1%) [3]. Recently, the tree peony seeds were identified as a novel source of edible oil with abundant UFAs (> 90%), and a high proportion of ALA (> 40%), which is much higher than many other plant oils [4–6]. Therefore, tree peony could be regarded as an excellent model for investigating the synthesis and production of ALA.

In plants, lipid accumulation mainly includes fatty acid (FA) synthesis and triglyceride (TAG) assembly. Particularly, sucrose is converted into pyruvate via glycolysis with pyruvate kinase (PK) catalyzing the final step, which provides precursors for fatty acid production [7]. Then, the acetyl-CoA is rapidly generated from pyruvate by the action of the plastidial pyruvate dehydrogenase complex (PDHC) to maintain the de novo fatty acid biosynthesis [8]. In the first step, the formation of malonyl-CoA from acetyl-CoA was catalyzed by the biotin carboxyl carrier protein (BCCP) [9]. Then, malonyl-CoA was converted to malonyl-ACP with the action of malonyl-CoA:ACP transacylase (MCAT) and reduced by enoyl-ACP reductase (ENR). After the series of subsequential condensation reactions driven by 3-ketoacyl-ACP synthases isoform (KAS), 16:0-ACP or 18:0-ACP were formed and hydrolyzed by thioesterases (FATA/FATB) for export from the plastid to the acyl-CoAs pool [10]. In the endoplasmic reticulum (ER), acyl-CoAs are used for the sequential acylation of glycerol-3-phosphate (G3P) backbone to produce TAGs either by Kennedy pathway, including G3P acyltransferase (GPAT), lysophosphatidic acid acyltransferase (LPAT), and diacylglycerol acyltransferase (DGAT) or by acyl exchange from phosphatidylcholine (PC) to diacylglycerol (DAG) by the phospholipid: diacylglycerol acyltransferase (PDAT) [11–13].

Transcription factors (TFs) such as Leafy cotyledon 1 (LEC1), Leafy cotyledon 2 (LEC2), Abscisic and insensitive 3 (ABI3), FUSCA 3 (FUS3), and Wrinkled 1 (WRI1) also were found played an important role in regulating the biosynthesis of *Arabidopsis* seed oil [14–16]. One of the most important TFs is *WRI1*, which is located in the downstream of lipid regulation network and was recently identified as a target of KIN10, the major SUCROSE NON-FERMENTATION1-RELATED KINASE1 involved in sugar/energy homeostasis [15, 17]. *WRI1*, belonging to APETALA2 (AP2) transcription factor family, was first identified in the *Arabidopsis* mutant line with wrinkled seed and 80% fewer TAGs compared to the wild type [18]. The genes regulated by *WRI1* were involved in glycolysis, lipoic acid, and FA biosynthesis pathways [19]. And, it has been demonstrated that *WRI1* is able to bind to the promoters of genes encoding key enzymes including biotin carboxyl carrier protein isoform 2 (BCCP2), acyl carrier protein 1 (ACP1), and keto-ACP synthase 1 (KAS1), which provide precursors (acyl chain and glycerol backbones) for various lipid biosynthetic pathways [20, 21]. And, the overexpression of *AtWRI1*or its orthologs from rapeseed (*Brassica napus* L.), corn (*Zea mays* L.), and oil palm (*Elaeisguineensis Jacq*), has led to varying degrees of increases in oil accumulation of seeds [22–24]. And, the recent findings showed that *WRI1* is the key regulator of oil biosynthesis in *P. ostii* developing endosperm [25, 26]. However, the role of *WRI1* during the accumulation of *P. rockii* seeds oil has not been uncovered.

In the present study, we investigated the role of *WRI1* in the seeds of *P. rockii* with high UFAs. Its expression patterns in different tissues and seed development stages were also analyzed. Further, *PrWRI1* was cloned and characterized by transient expression in the leaves of *Nicotiana benthamiana* and *Arabidopsis* using a stable transformation approach*.* Over-expression of *PrWRI1* in *Arabidopsis* increased the total fatty acids, mainly UFAs in the seeds. Furthermore, the transcript level of genes involved in acyl editing and transfer pathways also increased in the transgenic *Arabidopsis*. Identification and characterization of the *PrWRI1* would be meaningful for the genetic improvement of oil crops and could also lay foundations for the synthetic biology of FAs.

## Results

### Isolation and structural analysis of PrWRI1

**Fig. 1:**
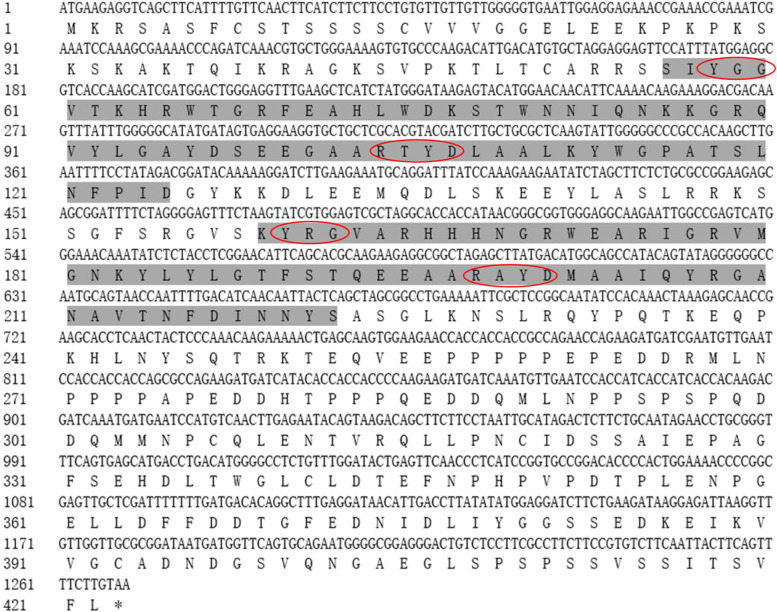
Deduced amino acid sequences of *PrWRI1* proteins and conserved domain analysis. The two parts in gray background indicate the two conserved DNA-binding domains (BDs) (AP2/ERF domain), and the elliptical frame indicates BDs

The full-length cDNA sequence of *PrWRI1* was identified by sequence similarity search of the *AtWRI1* encoding sequence against the *P. rockii* transcriptome assembly and named as *PrWRI1*. The ORF of *PrWRI1* consisted of 1269 nucleotides, and it encodes a putative protein of 422 amino acids with a predicted molecular mass of 46.93 kDa and a pI of 5.52. The deduced amino acid sequence analysis showed that this protein had two typical AP2/ERF DNA-BD at 56–125 amino acid (aa) and 159–222 aa. Additionally, *PrWRI1* has conserved YRG and RAYD residues in two AP2/ERF domains, which suggests that *PrWRI1* might belong to the AP2 subfamily (Fig. 1).

**Fig. 2:**
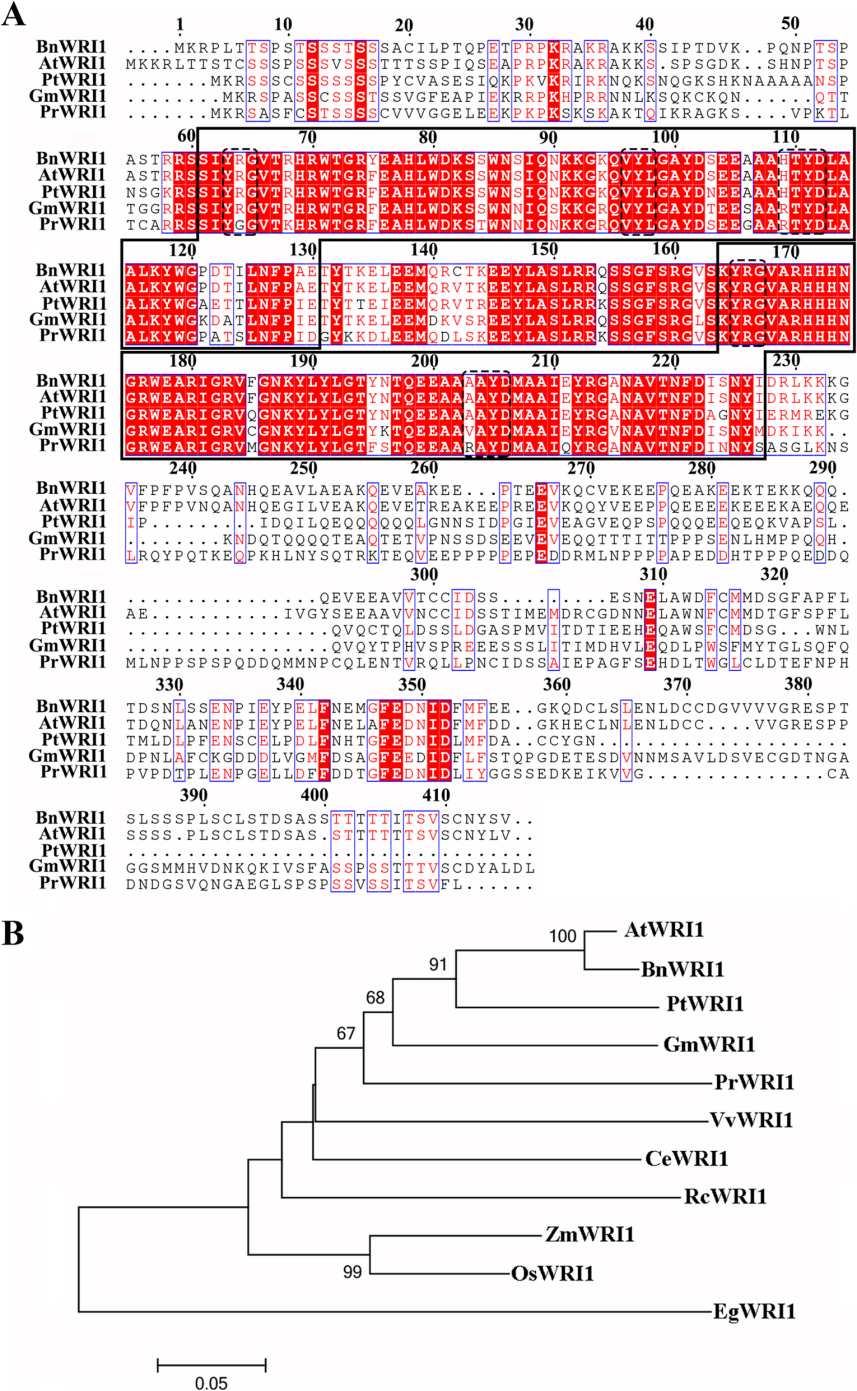
**A** Comparison of the deduced amino acid sequences for AP2/ERF-related proteins that have high sequence similarity with *PrWRI1*. Amino acids that are the all same among five different species are shown in red background. The conserved signature motifs are highlighted by blue boxes. The AP2/ERF domains were marked with black boxes. **B** Phylogenic comparison of the *PrWRI1* protein and some AP2/ERF-related protein sequences based on the selected AP2/ERF domain amino acid sequences for those proteins

There is a high identity in the conserved AP2 domains of *PrWRI1*, when compared with those of *BnWRI1* (ADO16346.1), *AtWRI1* (AAP80382.1), *PtWRI1* (XP_002311921.2), and *GmWRI1* (XP_006596986.1). But, the sequences at the C-terminal regions are diverged (Fig. 2A). Phylogenetic analysis indicated that the *PrWRI1s* are classified into the same group with those of *Arabidopsis* and *B. napus* among the various *WRI1s* that are currently identified from plants (Fig. 2B). And, *PrWRI1* has a much closer relationship with *GmWRI1* (soybean).

### PrWRI1 expression in different tissues and developing seeds

**Fig. 3:**
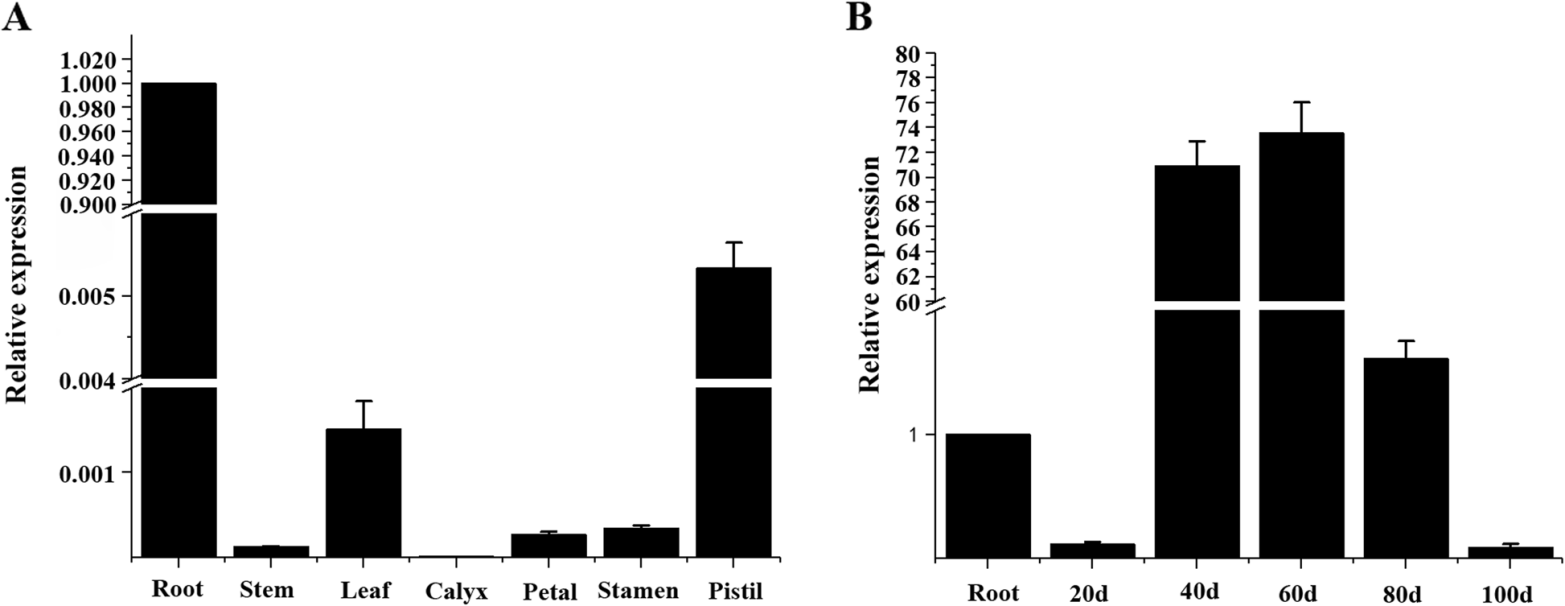
Expression patterns of *PrWRI1* in different tissues **A** and developing seeds **B** of *P. rockii*. The expression abundance was calibrated relative to WRI1 expression level in the roots of *P. rockii*. Values are expressed as means ± SD

To gain further insights to the organ-specific expression of *PrWRI1*, the expression patterns of *PrWRI1* in different tissues of tree peony were determined using quantitative Real-time PCR (qRT-PCR). In non-seed tissues, *PrWRI1* was higher expressed in root and pistil than in others (Fig. 3A). As shown in Fig. 3B, the transcript abundances of *PrWRI1* in developing seeds were much higher than that of roots in *P. rockii*. The *PrWRI1* was markedly activated, reached a maximum expression level at 40 and 60 DAP, and then gradually decreased throughout the seed maturation. Therefore, the transcription factor *PrWRI1* might mainly function during tree peony seed development period.

### Subcellular localization of the PrWRI1 protein

**Fig. 4:**
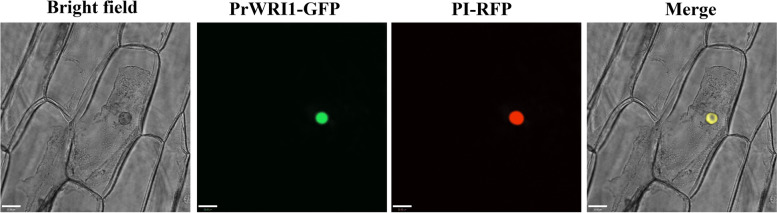
Subcellular localization of the *PrWRI1* protein fused with GFP (35S: *PrWRI1-GFP*) in onion epidermal cells. Bars = 33 µm

To further detect the subcellular localization of *PrWRI1*, the *35S::PrWRI1-GFP* translational fusion protein was generated and transiently transformed with the marker pBV220-cherry into onion inner epidermal peels by *Agrobacterium*-mediated transformation method. The results showed that the red fluorescence of pBV220-mCherry was merged with the green fluorescence of *35S::PrWRI1-GFP* in the nucleolus, indicating the subcellular localization of *PrWRI1* to the nucleolus (Fig. 4).

### Transient overexpression of PrWRI1

**Fig. 5:**
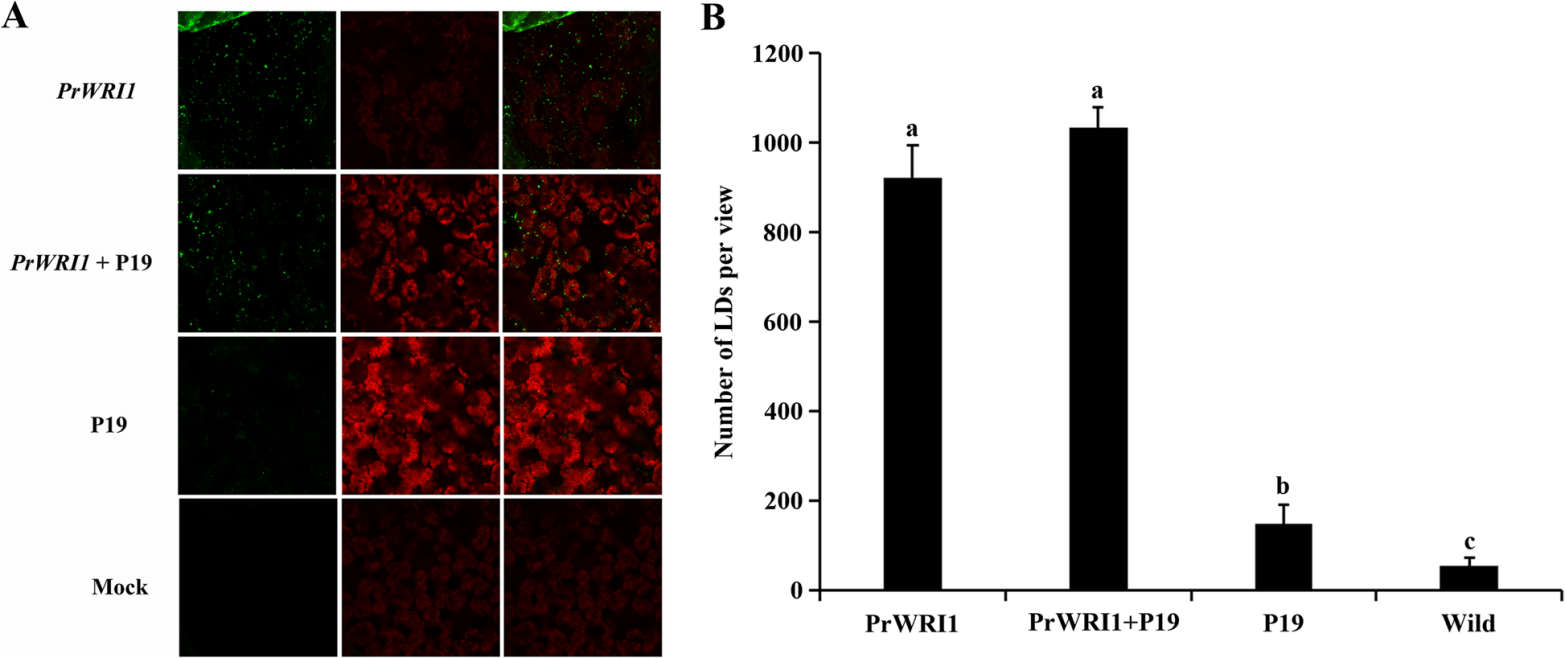
**A** Representative confocal images of LDs in *N. benthamiana* leaf tissue. Green color shows LDs and red color shows chloroplast. **B** Number of total LDs per image area in *PrWRI1*-transformed, mock, and P19-transformed *N. benthamiana* leaf tissue

As an advantageous transient expression system, the *N. benthamiana* leaf tissue also has been successfully applied to characterize the functions of genes involved in FA biosynthesis. The *PrWRI1* was over-expressed under the control of dual CaMV 35S promoters in *N. benthamiana* leaves. The Tomato bushy stunt virus (TBSV)-encoded p19 protein (P19) was also co-transformed as an inhibitor of ectopic gene silencing. Six days after infiltration, the lipid droplets (LDs) in leaves were observed under the confocal fluorescence microscope. According to the results, the number of LDs was significantly increased in *PrWRI1-*overexpressed leaves, when compared with that in mock- and P19-transformed control leaves (Fig. 5). And, when compared to the leaves that had just been transformed with *PrWRI1*, more LDs were found in leaves that had also been transformed with P19.

### Generation of transgenic Arabidopsis and gene expression analysis

In order to investigate the role of *PrWRI1* in the stable genetic transformation system, a plant over-expression vector harboring *PrWRI1* driven by the 35S promoter was constructed and transformed into wild-type *Arabidopsis* (Columbia-0). In total of five independent T3 homozygous transgenic lines with high expression of *PrWRI1* were identified by RT-PCR for further analysis.

**Fig. 6:**
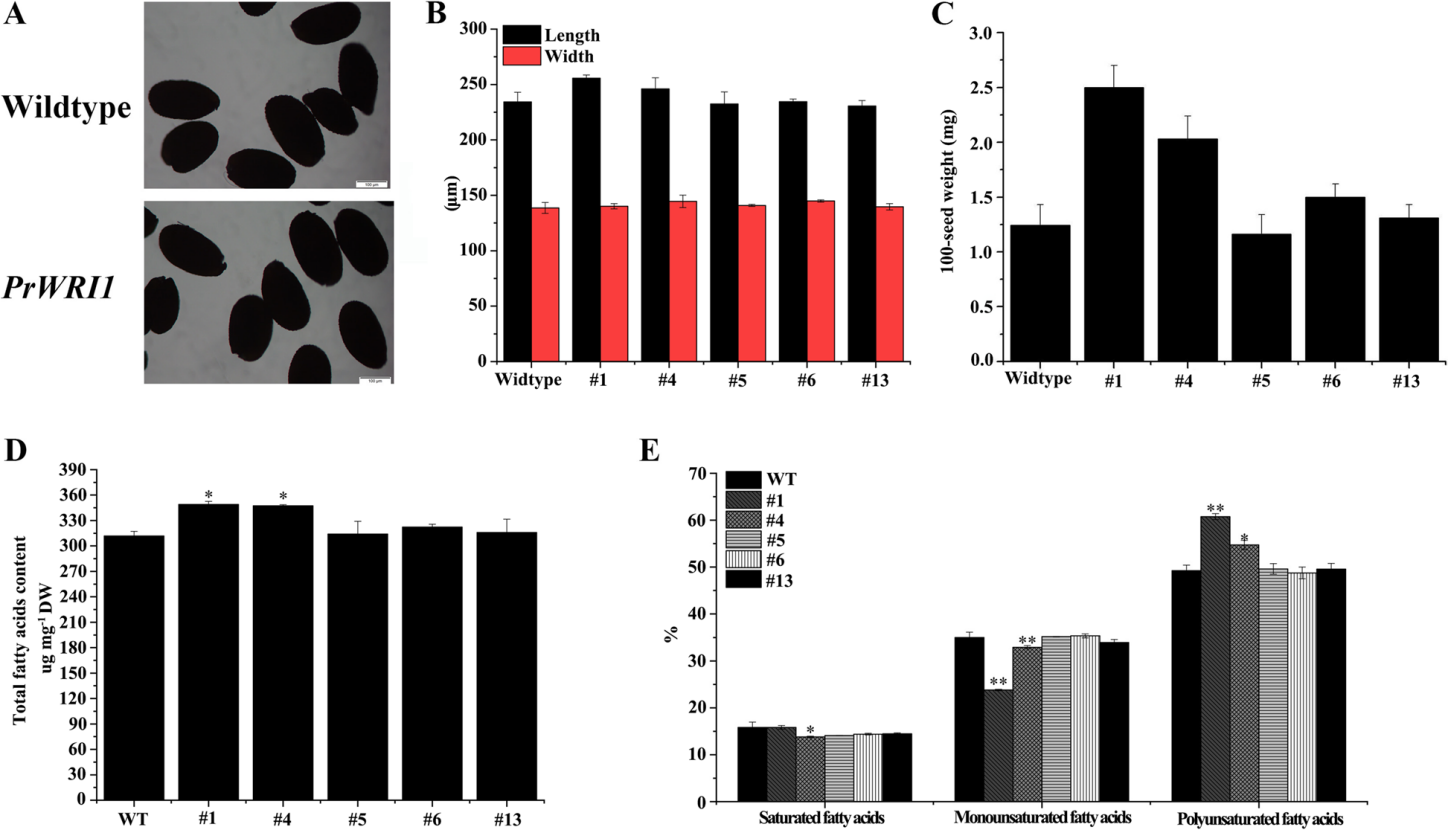
Effect of *PrWRI1* overexpression on the phenotype **A**, length, width **B**, weight **C**, FA content **D** and composition **E** of seed from independent homozygous T3 lines. Values are expressed as mean ± SD (*n* = 3). Star symbols indicate significant difference at *P* < 0.05

The length and width of seeds from T3 transgenic lines and wild-type were measured. The seeds of transgenic *Arabidopsis* are plumper in shape, and larger in 100 seeds weight, when compared with wild-type ones (Fig. 6A, B and C). The average weight of 100 transgenic seeds was 1.72 mg, which was 37.6% higher than that of wild seeds. Furthermore, the total fatty acid content in transgenic lines (#1 and #4) was significantly higher than that in wild-type (Fig. 6D). And, the relative abundance of PUFAs were significantly increased, while the proportion of monounsaturated fatty acids were declined in #1 and #4 transgenic lines (Fig. 6E). Overall, these results suggested that *PrWRI1* might play an important role in enhancing oil accumulation and changing FA composition.

**Fig. 7:**
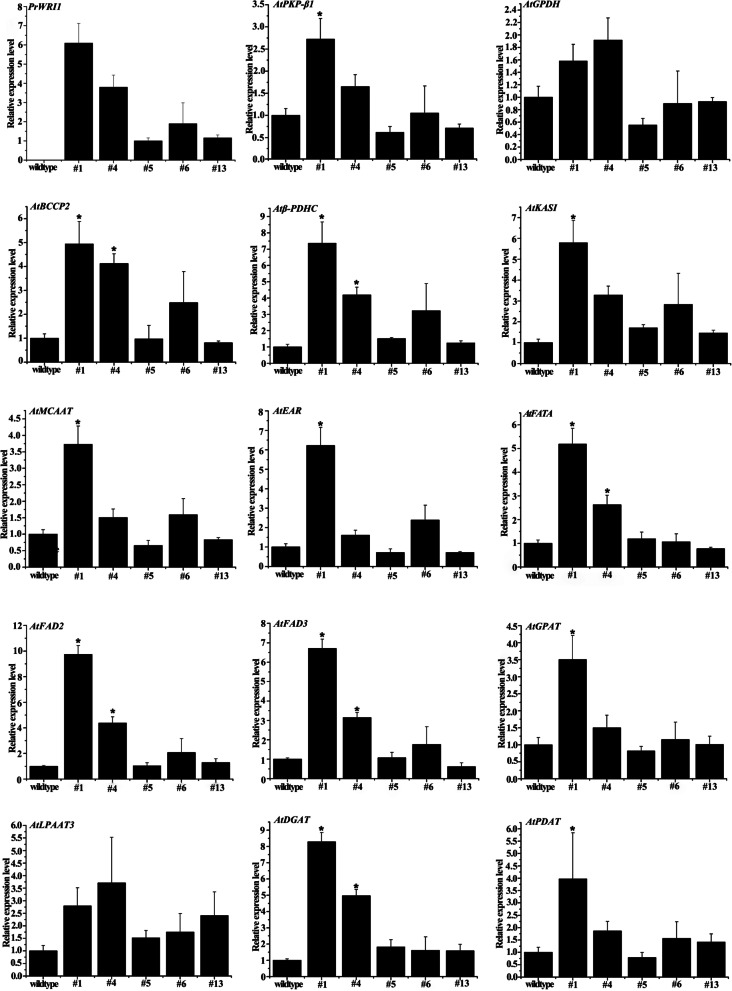
Expression analysis of genes involved in the FAs and TAG biosynthesis pathway in mature seeds from the homozygous transgenic *PrWRI1 Arabidopsis* seeds. Values are expressed as mean ± SD (*n* = 3). Star symbols indicate significant difference at *P* < 0.05

Furthermore, the expression of various FA and TAG biosynthesis genes (including *PKP-β1*, *GPDH*, *BCCP2*, *β-PDHC*, *KASI*, *MCAAT*, *EAR*, *FATA*, *FAD2*, *FAD3*, *GPAT*, *LPAAT*, *DGAT*, *PDAT*) in transgenic *Arabidopsis* seeds were also examined by qRT-PCR. The expression levels of genes related to pyruvate synthesis in glycolysis (*PKP-β1*), FA de novo synthesis (BCCP2, β-PDHC, FATA), FA desaturation (FAD2, FAD3), and TAG assembly (DGAT, PDAT) were increased diversely in #1 and #4 transgenic line seeds (Fig. 7).

## Discussion

*WRI1* was the key transcription factor regulating fatty acid biosynthesis and was first identified in *Arabidopsis* [27]. And, its orthologs have been identified in many plants including *Brassica napus* [22], *Glycine max* [28], *Ricinus connunis* [29], and *Zea mays* [30]. In the present study, the *PrWRI1* gene was identified and cloned from the seeds of *P. rockii*, which was characterized by high ALA content. The analysis of amino acid sequences showed that *PrWRI1* had conserved YRG and RAYD residues, which were proposed to be functional in DNA binding, and might belong to the AP2 TF’s family [31]. And, comparison of WRI1 orthologs across many diverse plant species revealed a short protein sequence “VYL” also presented in the first AP2 domain of *PrWRI1* (Fig. 2A). Studies have shown that the impairment of the function of *WRI1* protein in *Arabidopsis* could be resulted by the mutation of a single amino acid in “VYL”, suggesting the essential role of “VYL” for *AtWRI1* function [24]. However, recent studies have shown that the functional importance of "VYL" is in question because it is absent from AsWRI1c, RcWRI1-B, and OsWRI1-1 [29, 32, 33]. With the identification of AtWRI1 with its cognate double-stranded DNA, the mechanism by which WRI1 works will be further understood [34]. In addition, although the C-terminal regions of *PrWRI1* are diverged, bioinformatics analysis showed that *PrWRI1* has much closer relationship with *GmWRI1* (soybean), whose downstream genes are responsible for fatty acid synthesis, elongation, and desaturation [28].

During the development of *P. rockii* seeds, the content of fatty acid is relatively low in the immature seeds, then there is a period of rapid oil accumulation, and finally, the FA content enters into a relatively stable period with a slight decrease [35]. The expression levels of *PrWRI1* in immature seeds (40 and 60 DAP) were much higher than those in other periods during the development of *P. rockii* seeds (Fig. 3B). The results are in agreement with those in many other plants, such as coconut [36] and camelina [37]. That indicated *PrWRI1* is actively transcribed during seed development, especially during the fore and middle periods. Besides, the expression level of *PrWRI1* was much higher in roots than in other non-seed tissues in *P. rockii*. The relatively high expression level in *P. rockii* roots points to a root-specific function. In *A. thaliana*, WRI1 has been shown to have a role in root auxin homeostasis by controlling the expression of auxin carrier genes [38].

In the studies of transient overexpression of *PrWRI1*, the tomato bushy stunt virus (TBSV)-encoded p19 protein (P19) was also co-transformed as an inhibitor of ectopic gene silencing [39]. The P19 protein of TBSV is involved in various important activities, including the suppression of posttranscriptional gene silencing, virus movement, and symptom induction [40]. And, P19 was often applied to enhance heterologous gene expression with harmless to plants [41]. According to the results, the number of LDs was much more in *PrWRI1* and P19 co-overexpressed *N. benthamiana* leaves than that in mock- and P19-transformed control leaves, which indicates that P19 was an effective tool in the investigation of genes associated with lipid accumulation. Ectopic expression of *WRI1s* from castor and oat were also found to enhance TAG levels in *N. benthamiana* fresh leaves relative to the control [29, 33]. In addition to this, the visualizations of LDs in *N. benthamiana* leaves will be greatly helpful for the investigation of TAG accumulations, which also have been used in our other study on *PrASIL1* [42]. And, our results would provide a promising strategy to increase the production of vegetable leaves oils to meet the increasing demand for edible oil [43].

*WRI1* could regulate the metabolic processes, particularly glycolysis, during seed development [44]. The overexpression of *WRI1* alters the expression of target genes involved in glycolysis and fatty acid synthesis, and further enhancing the carbon flow from glycolysis to fatty acid synthesis in seeds and finally increasing the accumulation of seeds oil. The present study showed that the average weight of 100 *PrWRI1-*overexpressed transgenic *Arabidopsis* seeds was heavier than that of wild seeds. Similarly, ectopic expression of *BnWRI1-1* and *BnWRI1-2* from *Brassica napus* could significantly increase seed weights by around 40%, while the morphology of seeds was similar among transgenic lines and wild-type *Arabidopsis* [22]. This might have resulted from the increase in the sizes of cells and cotyledons in the seeds of transgenic lines. Besides, the content of total FA and PUFAs were also enhanced in transgenic lines (#1 and #4), when compared with that in wild-type. Similar results were also observed in the seeds of transgenic soybean lines overexpressing *GmWRI1a* gene [28]. The increased proportion of PUFAs, including C18:2 and C18:3, was also observed in transgenic *Arabidopsis* overexpressing *PoWRI1* from *Peasonia ostii* [45]. In sum, *WRI1* played an important role in enhancing oil accumulation and changing the FA composition of tree peony seeds oil.

In addition to phenotypic observation and FA measurement of transgenic *Arabidopsis* seeds, the expression levels of genes involved in glycolysis and fatty acid synthesis were also determined. According to the results, the transcript levels of genes related to FA de novo synthesis (*BCCP2*, *β-PDHC*, *FATA*) were increased, especially in transgenic lines (#1 and #4), which promotes the increase of raw materials for fatty acid synthesis and provides the material basis for fatty acid accumulation. As shown in Supplementary Table 3, a stronger correlation in the expression trends between WRI1 and genes relating with FA de novo was also observed in developing *P. rockii* seeds based on our previous transcriptome data [35]. Besides, the transcript levels of genes related to desaturation (*FAD2*, *FAD3*), and TAG assembly (*DGAT*, *PDAT*) were increased by varying degrees in transgenic *Arabidopsis* than those in wild-type ones. Taken together, these findings indicated that overexpression of *PrWRI1* could enhance the transcript levels of genes involved in the FA biosynthesis pathway, and consequently promote the accumulation of TAG in transgenic seeds. Similar results were also observed in over-expressed *CoWRI1* (*Cocos nucifera*) and *PoWRI1* (*Paeonia rockii*) transgenic *Arabidopsis* [36, 45]. The results indicated that the overexpression of *PrWRI1* not only promoted the flow of carbon source to FA metabolic pathway in transgenic *Arabidopsis* seeds, but also drove the expression of most genes related to FA biosynthesis.

## Conclusions

This investigation aimed to characterize *PrWRI1*, which has been isolated from *P. rockii* with high UFAs. *PrWRI1* had two typical AP2/ERF domains, and was similar to *GmWRI1* in structure. Besides, the expression patterns of *PrWRI1* in *P. rockii* suggested that *PrWRI1* was highly expressed in immature seeds. And, our results showed that *PrWRI1* over-expressed transient and stable system could increase total FAs and also change the FA compositions. Further, the expression of most genes related to FA synthesis and TAG assembly was increased in transgenic *Arabidopsis* seeds. Therefore, *PrWRI1* could push carbon flow to FA biosynthesis and further enhance the TAG amount in seeds. The mechanisms on the increased PUFAs in *PrWRI1-*overexpressed transgenic *Arabidopsis* seeds also need further investigation. Our results would provide a better understanding of *WRI1* transcriptional mechanisms in tree peony and could also be used in oil crops genetic research.

## Materials and methods

### Plant materials and growth conditions

Tree peony (*Paeonia rockii*) with the same genetic origin was grown in the wild tree peony germplasm repository at Yangling, Shaanxi Province, China. They were identified by Professor Li-xin Niu from Northwest A&F University. The voucher specimens of *Paeonia rockii* were deposited into the Herbarium of the National Oil Peony Engineering Technology Research Center, China. Different tissues including the root, stem, leaf, calyx, petal, stamen, pistil, and developing seeds (20, 40, 60, 80, and 100 days after flowering) were collected for transcript level analysis. All the samples were immediately frozen in liquid nitrogen and stored at -80 ℃ for further studies. *Arabidopsis thaliana* (ecotype Columbia-0) and *Nicotiana benthamiana* plants used for transformation were grown in growth chambers at 21/25 ℃ (day/night) with a 16 h light/8 h dark.

### Gene identification and isolation

Total RNA was extracted from the seeds collected at 20 days after pollination (DAP) according to the protocol of TIANGEN RNA Prep Pure Plant Kit (Tiangen Biotech Co. Ltd., Beijing, China). A full-length cDNAs library was constructed by using the PrimeScript® RT Reagent Kit with gDNA Eraser (Takara, Japan). The specific primers (Supplementary Table 1) for identifying the open reading frame (ORF) of the *PrWRI1* gene in tree peony were designed based on transcriptome data [35]. The *PrWRI1* was amplified and ligated into the pMD19-T vector and then sequenced.

### Protein sequence and phylogenetic tree analysis

The nucleotide and amino acid sequence analysis were performed using DNAMAN software. Homology search was conducted using the BLAST server in National Center for Biotechnology Information (NCBI). Dendrograms for phylogenetic analysis were performed on MEGA (version 5.1) software and multiple sequence alignment was conducted using CLUSTALW.

### Quantitative RT-PCR (qRT-PCR) analysis

Total RNA from various tissues of *Paeonia rockii* and *Arabidopsis* seeds (15 days after pollination) were isolated for transcript expression. The extraction of total RNA and the synthesis of cDNA were performed according to the method described above. The qRT-PCR was performed using SYBR® Premix Ex TaqTM (Perfect Real Time) kit (Takara, Dalian, China) in StepOnePlus Real-time PCR System (Applied Biosystems). Primers used for qRT-PCR were listed in Supplementary Table 2. The PCR reaction and data analysis were conducted according to the methods described previously [46]. All qRT-PCR experiments were performed in triplicate for each gene.

### Subcellular localization

The *PrWRI1* ORF without the stop codon was inserted into vector P2300-GFP using *BamHI* and *SalI* sites, generating *35S::PrWRI1-GFP* construct. Then, the constructed plasmid and the maker pBV220-mCherry were transformed into *Agrobacterium tumefaciens* strain EHA105 by electroporation, followed by the infection of onion inner epidermal peels using the agroinfiltration method (Horsch et al., 1984). The transformed cells were incubated for 24 h at 25℃ in the dark and the fluorescence was monitored using a laser scanning confocal microscope (Leica TCS SP8). The excitation wavelengths were 488 nm for GFP and 561 nm for markers.

### Vector construction

In order to investigate the activity of *PrWRI1* visually, the coding sequence of *PrWRI1* was cloned into the *SacII* and *BamHI* restriction sites of pK34 entry vector, and then the recombinant pK34 vector with double CaMV 35S promoters and a terminator sequence was digested with *AscI* for entry into plant expression vector, pB110. The vector was then transiently expressed in *Nicotiana benthamiana* leaves by Agrobacterium-mediated transformation.

The ORF of *PrWRI1* were inserted into the vector pCAMBIA1300 under the control of *Arabidopsis* seed-specific promoter 2S2 by the digestion of *KpnI* and *BamHI*. The generated constructs were transformed into *Agrobacterium tumefaciens* GV3101 using the freeze–thaw method, and then they were used for the transformation of wild-type *Arabidopsis* by the floral dip method.

### Transient expression in tobacco leaves

Five/six-day-old tobacco (*N. benthamiana*) leaves were chosen for infiltration. The pB110-*PrWRI1* construct was transiently transformed in *N. benthamiana* leaves individually or with the viral silencing suppressor protein P19 [47]. After infiltration, tobacco was transferred into the growth chamber and allowed to grow for 6/7 days to express the protein.

Then leaf discs were collected and placed into the Falcon tubes containing 4% formaldehyde in 1X phosphate buffered saline (PBS). The samples were washed three times with 1X PBS, after being shaken at 75 rpm for one hour. Finally, the leaf discs were stained with 4 μg/ml Nile Red in 1X PBS at room temperature in a rotational shaker at 100 rpm for 15 min in the dark. Then, each leaf disc was observed immediately under the confocal fluorescence microscope (Leica TCS SP8). The excitation wavelength for Nile Red is 488 nm and the emission wavelength is 560 to 620 nm.

The total number of lipid droplets (LDs) was counted by ImageJ software. Six biological replicates were conducted for each expression vector. Leaves infected with the empty vector or P19 vector were sampled as controls.

### Generation of transgenic Arabidopsis

The harvested seeds from transformed *Arabidopsis* plants were selected on 1/2 MS plates containing 20 mg/L hygromycin (hyg). Hyg-resistant seedlings were then transplanted into the moistened potting soil as *T*_*0*_ transformants and followingly confirmed by PCR analysis. Seeds from homozygous T3 transgenic lines with 100% hyg resistance were collected for further studies.

### Determination of seed weight and seed size

The seeds from wide-type and transgenic *Arabidopsis* lines were randomly counted and weighed using a microbalance. To determine the seeds’ sizes, they were examined and photographed using a Leica KL2 microscope (Leica Microsystems, Germany). The length and width of the seeds were measured using the program Image J (http://imagej.nih.gov.zzulib.vpn358.com/ij/) in accordance with the software's instructions.

### FA analysis

FA extraction and methylation were conducted according to the procedures described previously (Li et al., 2015). Gas chromatograph-mass spectrometer (Thermo Scientific trace 1310 GC-ISQ) and TriPlus RSH robotic sampler (Thermo Scientific) were used to analyze FAs. The helium was used as carrier gas in the TG-WaxMS capillary column (30 m × 0.25 mm internal diameter, 0.25 μm film thickness; Thermo Fisher Scientific, USA). Qualitative FA analysis was achieved using tridecylic acid as an internal standard. The FAs content was expressed as milligrams per gram dry weight (DW) of a sample. All samples were analyzed in triplicate.

### Statistical analysis

All experiments were performed in three biological replicates. The results were expressed as mean values ± standard deviations (SD). The significance of the difference between WRI1-overexpressed lines and wild-type was analyzed using One-way ANOVA. Statistical analysis was conducted using SPSS software (version 22.0 for Windows). All figures were generated using Origin 8.0 (Origin Software, Inc., OriginLab, USA).

## Supplementary Information

**Additional file 1:** **Supplementary Table 1.** Primers used for gene isolation and vector construction in the present study. **Supplementary Table 2.** Primers used for qRT-PCR analysis in the present study. **Supplementary Table 3.** The correlation analysis on the expression trends between WRI1 and other genes relating to fatty acid biosynthesis in developing P. rockii seeds based on the transcriptome data.

## Data Availability

All relevant data are included in the manuscript and its supporting materials.

## Abbreviations

FA: Fatty acid
UFAs: Unsaturated fatty acids
PUFAs: Polyunsaturated fatty acids
ALA: *α*-Linolenic acid TAG: Triglyceride
PK: Pyruvate kinase
PDHC: Pyruvate dehydrogenase complex
BCCP: Biotin carboxyl carrier protein
MCAT: Malonyl-CoA:ACP transacylase
ENR: Enoyl-ACP reductase
KAS: 3-Ketoacyl-ACP synthases isoform
FATA/FATB: Thioesterases
ER: Endoplasmic reticulum
G3P: Glycerol-3-phosphate
GPAT: G3P acyltransferase
LPAT: Lysophosphatidic acid acyltransferase
DGAT: Diacylglycerol acyltransferase
PC: Phosphatidylcholine
DAG: Diacylglycerol
PDAT: Phospholipid: diacylglycerol acyltransferase
TFs: Transcription factors
LEC1: Leafy cotyledon 1
LEC2: Leafy cotyledon 2
ABI3: Abscisic and insensitive 3
FUS3: FUSCA 3
WRI1: Wrinkled 1
AP2: APETALA2
aa: Amino acid
qRT-PCR: Quantitative real-time polymerase chain reaction
TBSV: Tomato bushy stunt virus
LDs: Lipid droplets
DAP: Days after pollination
DW: Dry weight
