# Supplementary material for: Tree peony transcription factor PrWRI1 enhances seed oil accumulation
Source: BMC Plant Biol. 2023 Mar 7;23:127. doi: 10.1186/s12870-023-04127-9 (PMC9990299; doi:10.1186/s12870-023-04127-9)
Supplement: Supplementary file 1 — Additional file 1: Supplementary Table 1. Primers used for gene isolation and vector construction in the present study. Supplementary Table 2. Primers used for qRT-PCR analysis in the present study. Supplementary Table 3. The correlation analysis on the expression trends between WRI1 and other genes relating to fatty acid biosynthesis in developing P. rockii seeds based on the transcriptome data. [file 12870_2023_4127_MOESM1_ESM.docx]

**Supplementary Table 1** Primers used for gene isolation, vector construction and transgenic confirmation in the present study.

| **Primer name** | **Primer sequence (5’-3’)** |
| --- | --- |
| *PrWRI1*-F | GAGGAGGAGGAGGAAGTAG |
| *PrWRI1*-R | TACACGAGCATAAGCCCAC |
| *35S:PrWRI1-GFP*-BamHI-F | CGGGATCCCGATGAAGAGGTCAGCTTCATTT |
| *35S:PrWRI1-GFP*-SalI-R | GCGTCGACCAAGAAAACTGAAGTAATTGA |
| SacⅡ-PrWRI1-F | TCCCCGCGGATGAAGAGGTCAGCTTC |
| BamHI-PrWRI1-R | CGCGGATCCTTACAAGAAAACTGAAGTAATTG |
| *35S:PrWRI1*-KpnI-F | GGGGTACCCCATGAAGAGGTCAGCTTCATTT |
| *35S:PrASIL1*-BamHI-R | CGGGATCCCGTTACAAGAAAACTGAAGTAAT |
| 1300-F | GGCTTTACACTTTATGCTTC |
| *PrWRI1-*RTR | TCAGTATCCAAACAGAGGC |

**Supplementary Table 2** Primers used for qRT-PCR analysis in the present study.

| **Primer name and accession numbers** | **Primer sequence (5’-3’)** |
| --- | --- |
| 18S-26S ITS | ACCGTTGATTCGCACAATTGGTCATCG |
|  | TACTGCGGGTCGGCAATCGGACG |
| *PrWRI1* | ATCACCATCACCACAAGAC |
|  | TCAGTATCCAAACAGAGGC |
| *AtPKP-β1* (AT5G52920) | AGTCACTATCGTCCTTCCG |
|  | CTGTACGATTGCTATTTCCTC |
| *AtGPDH* (AT2G41540) | GGGAGGTCTCAAGAATGTCTACGC |
|  | AGCAAAGGCCCTGCAAGTT |
| *AtBCCP2*(AT5G15530) | AACCCAATGGGATCTCCTTTCCCT |
|  | ATAAATTCAGAGAGCTCGGCGGGT |
| *Atβ-PDHC* (AT1G30120) | TGGAGCTGCCATGACTGGTCTA |
|  | TTTGGCGTTGTAAGGAGTTG |
| *AtKASI*(AT5G46290) | TCGATTTCAACTGCTTGTGC |
|  | CCTCCCAACCCAATAGGAAT |
| *AtMCAAT*(AT2G30200) | GCTGATTACAAACCCACCTC |
|  | GCAAGTCACATCAACCGAGT |
| *AtEAR*(AT2G05990) | TGGGACTTGGGTTCCTGCAC |
|  | CGCTTATTCGTTTTCACATCTTCAGGC |
| *AtFATA*(AT3G25110) | AGCTGATCTCGACATGAACCAGCA |
|  | ATTTCAGAGGTGGTGGTGGTGAGT |
| *AtFAD2*(AT3G12120) | ATGGGTGCAGGTGGAAGAAT |
|  | CCAGGAGAAGTAAGGGACGA |
| *AtFAD3*(AT2G29980) | CCACAGTACTCGGATGCTCAGA |
|  | GCAATAAGCTTTCTCTCGCTTGGA |
| *AtGPAT*(AT5G60620) | TCGGAAACCGGCGACGTAAGC |
|  | TGGCACCAGCAGCTTCAGTGAG |
| *AtLPAAT*(AT4G30580) | GGTCGCATTTCTAATGGCATGACG |
|  | TTCACTCGGGACAGCATGAAGG |
| *AtDGAT*(AT2G19450) | TCGCTCCCACATTGTGTTATCAGC |
|  | AAATTGACGAGCCACCCAACCC |
| *AtPDAT*(AT5G13640) | AAAGGATGTTGCAGTTGCCAGAG |
|  | TGTTGAGTCCCATGTGCGTGTC |
| *AtActin7*(At5g09810) | GGAACTGGAATGGTGAAGGCTG |
|  | CGATTGGATACTTCAGAGTGAGGA |

**Supplementary Table 3** The correlation analysis on the expression trends between WRI1 and other genes relating to fatty acid biosynthesis in developing *P.rockii* seeds based on the transcriptome data (Zhang et al., 2019).

|  | Correlation coefficient |
| --- | --- |
| PKP-β1 | 1.00 |
| GPDH | 1.00 |
| BCCP2 | 1.00 |
| β-PDHC | 0.84 |
| KAS I | 0.75 |
| MCAAT | 0.94 |
| EAR | 0.98 |
| FATA | -0.29 |
| FAD2 | -0.73 |
| FAD3 | -0.93 |
| GPAT | 0.98 |
| LPAAT | 0.65 |
| DGAT | -0.75 |
| PDAT | -0.83 |
